# Advances on Post-translational Modifications Involved in Seed Germination

**DOI:** 10.3389/fpls.2021.642979

**Published:** 2021-03-22

**Authors:** Feng Yu, Ming Li, Dongli He, Pingfang Yang

**Affiliations:** State Key Laboratory of Biocatalysis and Enzyme Engineering, School of Life Sciences, Hubei University, Wuhan, China

**Keywords:** post-translational modification, seed germination, phosphorylation, ubiquitylation, ABI5

## Abstract

Seed germination and subsequent seedling establishment are important developmental processes that undergo extremely complex changes of physiological status and are precisely regulated at transcriptional and translational levels. Phytohormones including abscisic acid (ABA) and gibberellin (GA) are the critical signaling molecules that modulate the alteration from relative quiescent to a highly active state in seeds. Transcription factors such as ABA insensitive5 (ABI5) and DELLA domain-containing proteins play the central roles in response to ABA and GA, respectively, which antagonize each other during seed germination. Recent investigations have demonstrated that the regulations at translational and post-translational levels, especially post-translational modifications (PTMs), play a decisive role in seed germination. Specifically, phosphorylation and ubiquitination were shown to be involved in regulating the function of ABI5. In this review, we summarized the latest advancement on the function of PTMs involved in the regulation of seed germination, in which the PTMs for ABI5- and DELLA-containing proteins play the key roles. Meanwhile, the studies on PTM-based proteomics during seed germination and the crosstalk of different PTMs are also discussed. Hopefully, it will facilitate in obtaining a comprehensive understanding of the physiological functions of different PTMs in seed germination.

## Introduction

Seed germination is an indispensable event for initiating seedling establishment and plant growth for next generation, which presents as an intricate physiological process precisely regulated by endogenous and environmental cues (Mazer, 1999; Donohue et al., 2005; Finch-Savage and Leubner-Metzger, 2006; Holdsworth et al., 2008). Numerous genes affecting seed germination have been cloned, mainly including *DOG1* (Nakabayashi et al., 2012; Graeber et al., 2014; Leubner-Metzger, 2014), *CYP707A1/2* (Millar et al., 2006; Matakiadis et al., 2009; Shu et al., 2013), *GA2oxs* (Yamauchi et al., 2007), *MYB96* (Lee H. G. et al., 2015; Lee K. et al., 2015), *OsAP2-39* (Yaish et al., 2010), *CHO1* (Yamagishi et al., 2009; Yano et al., 2009), *WRKY41* (Ding et al., 2014), and *ARF10/ARF16* (Liu et al., 2013). Almost all of these genes are involved in either abscisic acid (ABA) or gibberellic acid (GA) signaling, of which these two phytohormones play critical roles in seed germination through controlling the shift from seed dormancy to germination (Kucera et al., 2005; Finkelstein et al., 2008; Shu et al., 2016). The ABA molecule is recognized by its receptors PYRABACTIN RESISTANCE (PYR)/REGULATORY COMPONENT OF ABSCISIC ACID RECEPTOR (RCAR) (Ma et al., 2009; Miyazono et al., 2009; Nishimura et al., 2009; Santiago et al., 2009), and the ABA-bound receptors tightly combine with type 2C protein phosphatases (PP2Cs), resulting in the dissociation of SNF1-related kinases 2 (SnRK2s) from PP2C-SnRK2 complexes (Cutler et al., 2010). The released SnRK2s directly phosphorylate targeted transcription factors such as ABSCISIC ACID-INSENSITIVE5 (ABI5), ABI4, and ABI3 to mediate ABA responses (Kobayashi et al., 2005; Furihata et al., 2006; Fujii et al., 2007; Fujii and Zhu, 2009; Zhu et al., 2020). The mutant of ABA signaling related genes, such as biosynthetic genes aba deficient 1 (*aba1*) and nine-cis-epoxycarotenoid dioxygenase 6 (*nced6*) (Koornneef et al., 1982; Lefebvre et al., 2006), catabolic gene *cyp707a2* (Matakiadis et al., 2009) and signal transduction genes *abi3*, *abi4*, and *abi5* (Penfield et al., 2006; Park et al., 2011; Lim et al., 2013) in *Arabidopsis* had demonstrated that ABA could directly affect seed germination. GA, antagonizing with ABA, promotes seed germination (Holdsworth et al., 2008), which could be supported by the fact that GA-deficient mutants such as *ga1* and *ga2* fail to germinate (Lee et al., 2002; Shu et al., 2013). The balance between ABA and GA signal during seed germination is regulated by functional proteins such as GERMIN-LIKE PROTEIN 2-1 (OsGLP2-1), which binds to the promoters of *ABI5* and *GAMYB* (Wang et al., 2020) and INDUCER OF CBF EXPRESSION1 (*ICE1*) to antagonize ABI5 and DELLA activity (Hu et al., 2019).

Besides ABA and GA, other phytohormones, such as jasmonate (JA) (Pan et al., 2020), ethylene (Jurdak et al., 2020), cytokinin (Wang et al., 2011), auxin (Liu et al., 2013), and brassinosteroids (BRs) (Hu and Yu, 2014), also regulate the process of seed germination in *Arabidopsis*. JA regulates seed germination through ABA signaling. JA ZIM-DOMAIN (JAZ) proteins could inhibit the expression of *ABI3* and *ABI5* (Pan et al., 2020), whereas, JAZ repressors could physically interact with ABI3 and activate ABA signaling. The effect of ethylene on seed germination depends on the reactive oxygen specifies (ROS) molecules produced by the mitochondrial electron transport chain through up-regulating AOX1a and ANAC013 in mitochondrial retrograde response complex (Jurdak et al., 2020). The mutant of cytokinin biosynthesis exhibited ABA insensitive phenotype during germination, and the cytokinin signal transducers and transcription repressors, type-A ARR4, ARR5, and ARR6, could physically interact with ABI5 to negatively regulate ABI5 expression (Wang et al., 2011). The mutation of auxin signaling or biosynthesis in *Arabidopsis* dramatically released seed dormancy, which recruits auxin response factors (ARF) 10 and 16 to control the expression of *ABI3* during seed germination (Liu et al., 2013).

The synthesis, modification, localization, and degradation of proteins in the cells are critical for plants to survive from adverse environments, in which post-translational modifications (PTMs) of proteins increase the diversity of gene products and influence nearly every cellular process (Fulzele and Bennett, 2018). The prevalent PTMs mainly include phosphorylation (Mann et al., 2002; Ptacek et al., 2005; Thalassinos et al., 2008), ubiquitylation (Bennett et al., 2010; Xu et al., 2010; Kim et al., 2011), acetylation (Choudhary et al., 2009), glycosylation (Dell and Morris, 2001; Zhang et al., 2003), nitrosylation (Jaffrey et al., 2001), methylation (Clarke, 1993), and lipidation (Ichimura et al., 2000). With the development of analytical techniques, PTMs could be precisely detected at the global level or within a specific protein. For example, PHYTOCHROME INTERACTING FACTOR 3 (PIF3) is rapidly phosphorylated and degraded as a result of interaction between phytochrome B (phyB) and photo-activated PIF3, of which this process is needed for proper photomorphogenesis (Ni et al., 2013). Besides, the SUMOylated PIF3 in Lys13 residue also regulates the phyB abundant to affect plants’ photomorphogenesis (Bernula et al., 2020). PIF3 could be phosphorylated by multiple kinases such as GSK3-like kinase BRASSINOSTEROID-INSENSITIVE 2 (BIN2) and photo-regulatory protein kinases (PPKs), which is required for further ubiquitination of the proteins (Li et al., 2017; Xu et al., 2017).

**FIGURE 1:**
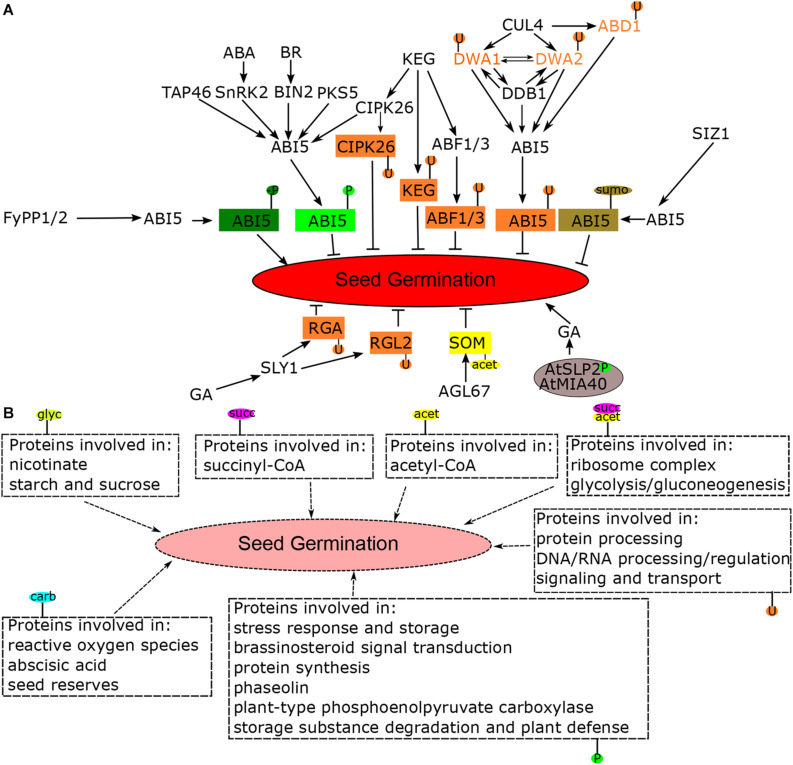
The diverse post-translation modifications (PTMs) involved in seed germination. **(A)** The key genes regulated by PTMs involved in seed germination. **(B)** The PTMs identified by proteomics techniques involved in seed germination. The phytohormones GA and ABA function antagonistically with each other in regulating seed germination, of which the signal transduction is largely coordinated by PTMs. ABI5, a key transcription factor in response to ABA, suppresses the seed germination, and it is phosphorylated by different kinases such as SnRK2, BIN2, CIPK26, PKS5, and KEG. KEG also ubiquitinates ABI5, CIPK26, and ABF1/3 to directly inhibit seed germination. DWA1, DWA2, and DDB1 interact with each other forming a complex that elevates the level of ABI5, in which DWA1 and DWA2 are ubiquitinated by CUL4. CUL4 also ubiquitinated ABD1 targeted for ABI5 degradation through 26S proteasome. The phosphatase FyPP1/2 and SIZ1 dephosphorylated and sumoylated ABI5, respectively. GA facilitated the expression of SLY1, which ubiquitinated two DELLA containing proteins RGA and RGL2 to derepress for germination. MADS-box transcription factor AGL67 acetylated the promoter of zinc-finger protein SOM to promote its expression that inhibited seed germination, and AtMIA40 formed the complex with AtSLP2 to coordinate its phosphatase activity, which negatively regulated the GA-related process under seed germination. Furthermore, the PTMs including phosphorylation, ubiquitination, carbonylation, glycosylation, acetylation, and succinylation were also involved in multiple metabolism processes such as protein processing, ribosome complex, brassinosteroid signal transduction, and reactive oxygen species. GA, gibberellin; ABA, abscisic acid; ABI5, ABA insensitive 5; SnRK2s, SNF1-related kinases 2; BIN2, BRASSINOSTEROID-INSENSITIVE 2; CIPK26, Calcineurin B-like Interacting Protein Kinase 26; PKS5, SOS2-like protein kinase 5; KEG, KEEP ON GOING; ABF1/3: ABRE binding factor 1/3; DWA1/2, DWD hypersensitive to ABA1; CUL4, CULLIN4; DDB1, damaged DNA binding1; ABD1, ABA-hypersensitive DCAF1; SIZ1, SUMO E3 ligases; SLY1, F-box-containing proteins SLEEPY1; RGA, REPRESSOR OF ga1-3; RGL2, RGA-like 2; SOM, SOMNUS; AGL67, AGAMOUS-LIKE67; AtSLP2, *Arabidopsis Shewanella*-like protein phosphatase 2; AtMIA40, *Arabidopsis* mitochondrial oxidoreductase import and assembly protein 40; P, phosphorylation; U, ubiquitination; carb, carbonylation; glyc, glycosylation; acet, acetylation; succ, succinylation; sumo, sumoylation.

Seed germination is an important physiological alteration from quiet dormant status to active seedling establishment, in which a large number of processes are reprogrammed. Han and Yang (2015) have summarized the progress in the molecular mechanisms of seed germination among different species, including morphological changes, cellular and its related structure recovery, metabolic variations, and transcription activation. However, more and more evidences have demonstrated that PTMs also play critical roles in regulating seed germination. In this paper, we reviewed the recent investigations on PTMs involved in seed germination (Figure 1), which will help to understand the molecular mechanism of seed germination.

## Phosphorylation Regulation of Seed Germination

Protein phosphorylation and dephosphorylation is an important switch for the activity of proteins, and more than 75% of eukaryotic proteins are potentially phosphorylated, which control almost all the biological processes (Sharma et al., 2014). ABI5 plays central roles in the ABA signaling pathway, and its phosphorylation regulation among ABA signaling transduction is well characterized, which directly influence the seed germination. In the presence of ABA, the protein kinase SnRK2 could phosphorylate ABI5 and promote its stability, through which seed germination was inhibited (Kobayashi et al., 2005; Fujii et al., 2007; Nakashima et al., 2009). The BR receptor BIN2 enhances the downstream signaling of ABA through phosphorylating ABI5 to further regulate the seed germination (Hu and Yu, 2014). SOS2-like protein kinase 5 (PKS5) also phosphorylates ABI5 at Ser-42 to regulate seed germination in *Arabidopsis* (Zhou et al., 2015). Calcineurin B-like Interacting Protein Kinase (CIPK) 26 not only interacts with ABI1, ABI2, and ABI5 in *Arabidopsis*, but also phosphorylates ABI5 *in vitro*, through which CIPK26 enhances the sensitivity of seed germination (Lyzenga et al., 2013). The *Arabidopsis* RING-ANK family protein, KEEP ON GOING (KEG), contains a kinase domain with phosphorylation activity, and negatively regulates ABA signaling through interacting with ABI5 (Stone et al., 2006). The mutation of two PROTEIN PHOSPHATASE6 (*PP6*) genes, *FyPP1* and *FyPP2*, lead to the ABA hypersensitive phenotypes at seed germination and seedling growth in *Arabidopsis*, which directly interact with and dephosphorylate ABI5, acting antagonistically with SnRK2 kinases (Dai et al., 2013). Moreover, a protein phosphatase 2A-associated protein TAP46 could enhance the stability of ABI5 through binding to and phosphorylating ABI5 (Hu et al., 2014). These investigations collectively indicated that the phosphorylation and dephosphorylation of ABI5 is precisely regulated in the ABA-mediated signaling pathway, which further controls seed germination and seedling establishment. The recent investigation also showed that *Arabidopsis* Shewanella-like protein phosphatase 2 (AtSLP2) interacts with the mitochondrial oxidoreductase import and assembly protein 40 (AtMIA40) in the mitochondrial intermembrane space, which is required for the phosphatase activity of AtSLP2, and the complex of AtSLP2 and AtMIA40 negatively regulates the GA-related process during seed germination (Uhrig et al., 2017).

Besides the functional elucidation of phosphorylation in specific proteins, phosphoproteomics is a well way to globally identify the phosphorylated proteins in the tissues, through which numerous phosphorylation modified proteins involved in seed germination have been discovered. Systematical analysis of phosphorylated proteins in the embryo of germinating seeds at the early stage demonstrated that phosphorylation of proteins related to stress response and storage was gradually enhanced, and proteins involved in BR signal transduction were also phosphorylated while brassinolide treatment enhanced the ability of seed germination in rice, implying seed germination is possibly triggered by BR signal (Han et al.,2014a,b). A gel-free/label-free phosphoproteomics were conducted to detect phosphorylated nuclear proteins at the early stage of rice seed germination (Li M. et al., 2015). The results demonstrated that proteins related to protein synthesis were mainly phosphorylated with 29 proteins displaying significant changes in phosphorylation level over the period of imbibition. Phosphorylation analysis of phaseolin in the dormant and 4-day germinating bean seed of two different cultivars found that the phosphorylation levels of phaseolin were remarkably changed from the dormancy status to early germination stage (López-Pedrouso et al., 2014). The phosphorylation of multiple plant-type phosphoenolpyruvate carboxylase (PTPC) isoenzymes at their conserved N-terminal seryl site was also identified in sorghum during seed germination (Ruiz-Ballesta et al., 2016). Furthermore, the proteins involved in storage substance degradation and plant defense were also detected to be phosphorylated in wheat (*Triticum aestivum* L.) during seed germination (Dong et al., 2015), suggesting that protein phosphorylation participates in diverse metabolism processes in seed germination.

## Ubiquitination Regulation of Seed Germination

Ubiquitination is another most prevalent PTMs in plants, which widely involves in various pivotal processes including protein turnover, genomic integrity, signaling processing among others (He et al., 2020a). GA is a key phytohormone that promotes seed germination with DELLA proteins being the most important repressors of GA signaling (Dill et al., 2001; Lee et al., 2002; Tyler et al., 2004; Davière and Achard, 2016). The GA receptor GID1 has a higher affinity for GA4, which also interacts with DELLA proteins (Nakajima et al., 2006). In the presence of GA, the F-box-containing protein SLEEPY1 (SLY1) directly interacts with the GA receptor and DELLA protein REPRESSOR OF ga1-3 (RGA) through their C-terminal GRAS domain, which mediates the ubiquitination and the subsequent degradation of RGA to promote the GA signaling pathway (Dill et al., 2001, 2004; Ueguchi-Tanaka et al., 2005). The RGA-like 2 (*RGL2*) is expressed during the imbibition period and plays a critical role in inhibiting seed germination, which is also degraded through F-box protein SLY1 mediated ubiquitination (Lee et al., 2002; Tyler et al., 2004; Piskurewicz et al., 2008). Other DELLA proteins, including GA insensitive (GAI), RGA and RGL1, enhance the function of RGL2, and the far-red light repressed the seed germination through stabilizing GAI, RGA, and RGL2 (Cao et al., 2005; Piskurewicz et al., 2009). These investigations illustrated that ubiquitination of DELLA proteins is the key step in derepressing the inhibition of GA-signaling involved in seed germination in *Arabidopsis*.

The level of ABI5 is also regulated by ubiquitination during seed germination. The RING-type E3 ligase KEG is needed to maintain the low level of ABI5, of which ABI5 is a substrate of KEG for ubiquitination (Liu and Stone, 2013). KEG is also self-ubiquitinated and degraded through the 26S proteasome system to increase the ABI5 level in response to ABA (Liu and Stone, 2010), and CIPK26 is also ubiquitinated by KEG and degraded through 26S proteasome (Lyzenga et al., 2013). Two ABI5-related transcription factor, ABRE binding factor 1 (ABF1) and ABF3, also regulate seed germination, and the detailed investigations demonstrated that KEG could directly interact with and ubiquitinate ABF1 and ABF3 to regulated their protein abundance (Finkelstein et al., 2005; Chen et al., 2013). The mutant of two *Arabidopsis* DWD proteins DWA1 (DWD hypersensitive to ABA1) and DWA2 that are substrate receptors of CULLIN4 (CUL4) E3 ubiquitin ligases displayed delayed germination, which were involved in ABA signal transduction (Lee et al., 2010). DWA1, DWA2 and Damaged DNA Binding1 (DDB1) can directly interact with each other to form CUL4-based complexes that target and mediate the ubiquitination of ABI5. The members of DDB1-CUL4–associated factors (DCAFs) family that bind to DDB1 are also the substrate receptors of CUL4. ABA-hypersensitive DCAF1 (ABD1) belongs to DCAF1 family that interacts with DDB1, and the loss of ABD1 could result in ABA-hypersensitivity phenotypes during germination and seedling growth (Seo et al., 2014). ABD1 directly interacts with ABI5 and the degradation of ABI5 by the 26S proteasome was also suppressed in the ABD1 mutant lines. Collectively, protein ubiquitination through E3 ubiquitin ligases is an important PTM regulating the ABI5 levels in ABA signaling during seed germination.

Furthermore, the high throughput ubiquitylome using K-ε-GG antibody enrichment integrated with mass spectrometry has been developed to identify a large amount of ubiquitinated proteins in tissues (Fulzele and Bennett, 2018). The PR-619-treated (deubiquitylase inhibitor) rice seed displayed a delayed germination, and analysis of ubiquitylated proteins at 0, 12, and 24 h after imbibition in rice embryos has demonstrated that 2,576 lysine sites in 1,171 proteins were ubiquitylated and the differentially ubiquitinated proteins were mainly involved in the categories of protein processing, DNA and RNA processing/regulation related, signaling and transport, indicating that ubiquitination is a regulator involved in the multifaceted process during seed germination (He et al., 2020b).

## Other PTMS Regulation of Seed Germination

Phosphorylation and ubiquitination are the two prevalent PTMs that have been widely studied and shown to be involved in many physiological processes through regulating protein activity and levels. However, it has been shown that many PTMs other than these two, such as sumoylation, carbonylation, glycosylation, acetylation, and succinylation, are also involved in seed germination. Overexpression of small ubiquitin-related modifier 1/2 (AtSUMO1/2) exhibited increased levels of sumoylation and was less sensitive to ABA (Lois et al., 2003), while SUMO E3 ligases SIZ1 directly sumoylated ABI5 enhancing its stability in response to ABA in seed germination (Miura et al., 2009). MADS-box transcription factor AGAMOUS-LIKE67 (AGL67) recruits the histone mark reader EARLY BOLTING IN SHORT DAY (EBS) to form a complex that is necessary for histone H4K5 acetylation in the promoter of zinc-finger protein SOMNUS (*SOM*), which activates *SOM* expression, ultimately inhibiting seed germination under high-temperature stress (Li et al., 2020). The investigation of rice acetylated or succinylated embryonic proteins after 24 h imbibition using nano-LC-MS/MS has identified 699 acetylated sites from 389 proteins and 665 succinylated sites from 261 proteins, which covered nearly all aspects of cellular functions, with ribosome complex and glycolysis/gluconeogenesis related proteins being significantly enriched (He et al., 2016). The enzymes related to acetyl-CoA and succinyl-CoA metabolism were modified through acetylation or succinylation, respectively. The dynamic pattern of protein carbonylation in rice embryos during germination was also analyzed using sequential window acquisition of all theoretical fragment ion spectra (SWATH) method, and 288 carbonylated peptides corresponding to 144 proteins were identified, which mainly involved in maintaining the levels of reactive oxygen species, ABA and seed reserves (Zhang et al., 2016). The mutant of *Arabidopsis UGT74F2*, a glycosyltransferase, accumulates high levels of glycosylated nicotinate, and the mutant displayed decreased rates of seed germination, of which the germination drawback of the *ugt74f2* mutant could be fully recovered by overexpressing *UGT74F2* (Li W. et al., 2015). Moreover, N-glycosylation mapping of rice embryos during germination has discovered 242 glycosites from 191 unique proteins, and these N-glycosylated proteins were enriched in starch and sucrose metabolism pathway, which were predicted to interact with several BR signaling proteins, implying that N-glycosylation is involved in carbohydrate metabolism and BR signaling to regulate seed germination (Ying et al., 2017).

## Crosstalk Among PTMS Involved in Seed Germination

The direct evidence of crosstalk regulation for PTMs involved in seed germination are from the modification of ABI5. As mentioned above, the protein levels of ABI5 could be precisely regulated through multiple PTMs. For example, protein kinase SnRK2, BIN2, PKS5 promote phosphorylation of ABI5 (Kobayashi et al., 2005; Fujii et al., 2007; Nakashima et al., 2009;, Hu and Yu 2014, Zhou et al., 2015), whereas protein phosphatase FyPP1 and FyPP2 lead to dephosphorylation of ABI5 (Dai et al., 2013). In addition, DWA1, DWA2, and ABD1 directly interact with and ubiquitinate ABI5 (Lee et al., 2010; Seo et al., 2014), and the SIZ1 sumoylates ABI5 during seed germination (Miura et al., 2009), KEG protein contains RING-HC and kinase domains, which function in ubiquitination and phosphorylation activity of ABI5 to control its protein level, respectively (Stone et al., 2006; Liu and Stone, 2010, 2013). KEG also ubiquitinated CIPK26 that phosphorylates ABI5. These results demonstrate the existence of crosstalk among phosphorylation, ubiquitination and sumoylation, with ABI5 being the node. Moreover, N-glycosylation and N-acetylation sites were predicted at the N terminus of ABI5 although experimental evidence is needed (Yu et al., 2015). Acetylation and succinylation analysis of germinated embryos in rice identified 133 common sites on 78 proteins modified by these two PTMs (He et al., 2016), implying the potential crosstalk between acetylation and succinylation during seed germination. Of the ubiquitylome in germinated rice embryo (He et al., 2020b), 88 proteins were also modified by phosphorylation (Han et al., 2014a) and 82 lysine residues in 49 proteins were also modified by acetylation (He et al., 2016), of which 12 proteins were modified by these three PTMs, indicating that co-modification occurred in these proteins. However, detailed investigations of specific proteins or sites are needed to clarify the molecular mechanism of how these PTMs co-regulated seed germination.

## Concluding Remarks

A successful break of dormancy in seed to initiate germination is an irreplaceable process in the plant life cycle, and numerous efforts have been conducted to investigate the molecular mechanisms underlying the initiation of seed germination and seedling establishment at (post-)transcriptional and (post-)translational levels. This review presented here provides a relatively comprehensive summary of the PTMs involved in the regulation of seed germination, which mainly included phosphorylation, ubiquitination, sumoylation, carbonylation, glycosylation, acetylation, and succinylation. The PTMs on ABI5 provides a model that could be used to understand the stability and activity of specific proteins modified by different PTMs. However, numerous questions related to PTMs involved in seed germination still need to be investigated in the future. This is not only decided by the complexity of germinating processes but limited by the analytic techniques including the accuracy of identification and analysis for PTMs. Not only the protein functions are determined by the combination of multiple PTMs but also the crosstalk among PTM regulation of seed germination remains largely unknown. The summarization of PTMs presented here will promote the study of the molecular basis underlying seed germination especially for the processes regulated by PTMs. The future work will be mainly conducted to mine clues in regulation of seed germination as follows: (i) PTMs involved in the other hormones such as auxin and ethylene, except ABA and GA; (ii) whether phosphorylation or other PTMs are involved in GA signaling, because only ubiquitination was detected in DELLA proteins; (iii) the detailed mechanisms of PTMs such as sumoylation, carbonylation, glycosylation, acetylation, and succinylation identified through proteomics technique and how they regulate seed germination; and (iv) whether other PTMs are involved in seed germination since over 600 PTMs have been detected so far.

## Author Contributions

FY and PY contributed to conceptualization and original draft preparation. PY, DH, and ML involved in review and editing. All authors contributed to the article and approved the submitted version.

## Conflict of Interest

The authors declare that the research was conducted in the absence of any commercial or financial relationships that could be construed as a potential conflict of interest.
